# Posttraumatic secondary brain insults exacerbates neuronal injury by altering Metabotropic Glutamate Receptors

**DOI:** 10.1186/1471-2202-8-96

**Published:** 2007-11-17

**Authors:** Zhou Fei, Xiang Zhang, Hong-min Bai, Xiao-fan Jiang, Xia Li, Wei Zhang, Wei Hu

**Affiliations:** 1 Department of Neurosurgery, Xijing Hospital, Fourth Military Medical University, Xi'an, 710032, P.R.China; 2 Department of Neurosurgery, Guang Zhou Liuhuaqiao Hospital, Guang Zhou, 510010, P.R.China

## Abstract

**Background:**

Our previous studies indicated that metabotropic glutamate receptors (mGluRs) are deeply involved in the secondary processes after diffuse brain injury (DBI). In the present study, we used a rodent DBI model to determine whether hypotension exacerbates neuronal injury as a secondary brain insult (SBI) after traumatic brain injury (TBI) by changing the expression of metabotropic glutamate receptors (mGluRs) in the cerebral cortex.

**Results:**

Three hundred and eleven male Sprague-Dawley rats were randomly assigned into five groups: normal control, sham-operated control, SBI alone, DBI alone, or DBI with SBI. DBI was produced in rats by Marmarou's methods and the SBI model was produced by hypotension. The alteration of neuronal expression of mGluRs after DBI and DBI coupled with SBI was observed by hybridization in situ at different time points in the experiment. We found a higher mortality and neurological severity score (NSS) for rats in the DBI with SBI group compared with those in the DBI alone group. Although there was a significant rise in the expression of group I and group III mGluRs (except mGluR6) and a decrease in the expression of group II mGluRs after DBI (P < 0.05), the changes were more severe when DBI was coupled with SBI (P < 0.05). The expression of group I mGluRs peaked at 24 hours, while the expression of the group III mGluRs peaked at 6 hours after injuries, which may reflect a self-protection first mechanism of the damaged neurons. Moreover, the overall neuro-harmful effects of mGluRs on neurons were seemly associated with higher mortality and NSS in the DBI with SBI group.

**Conclusion:**

The results suggest posttraumatic SBI may exacerbate neuronal injury or brain injury by altering expression of mGluRs, and more emphasis should therefore be put on the prevention and treatment of SBI.

## Background

Traumatic brain injury (TBI) remains a major cause of morbidity and mortality, particularly in young people. In TBI, the injured brain may be damaged by primary impact, but secondary brain insults (SBI) following primary impact (e.g., secondary ischemic insults) are other important causes of damage to the brain [1,2]. In the past two decades, a great number of studies have been carried out on SBI. It is shown that SBI such as pre-hospital hypotension or hypoxia [3-8], intraoperative hypotension [9], and hypoxia and hypotension in the intensive care unit [4,10-12] is consistently associated with poor outcomes. Hypotension is one of the most significant SBI after head injuries [13]. The combination of hypotension and head injury is associated with increased mortality and morbidity in comparison with head injury alone [14,15]. Despite encouraging animal studies, however, human trials assessing the use of pharmacological agents after TBI have all failed to show efficacy. Therefore, current management strategies are directed towards providing an optimal physiological environment in order to minimize secondary brain insults and maximize the body's own regenerative processes.

Two general mechanisms have been suggested for the increased sensitivity of the traumatized brain to SBI. First, trauma induces cellular processes that make the brain more sensitive to an additional insult. Second, trauma impairs the ability of the brain to regulate cerebral blood flow (CBF) and results in secondary ischemic insults [16]. The underlying cellular processes seem to be related to many biological events. However, it is suggested that the metabotropic glutamate receptors (mGluRs) are involved in the complex pathophysiology of brain injury [17-19]. The mGluRs are a family of glutamate-sensitive receptors different from the ionotropic glutamate receptors (iGluRs). So far, eight mGluR subtypes (designated from mGluR1 to mGluR8) have been cloned from mammalian brain tissue, which are classified into three sub-groups: Group I mGluRs (mGluR1 and mGluR5), Group II mGluRs (mGluR2 and mGluR3) and Group III mGluRs (mGluR4, mGluR6, mGluR7 and mGluR8) [20-23]. We hypothesize that mGluRs may also be involved in the cellular processes that make the brain more sensitive to an additional insult. To determine whether mGluRs are involved in the cellular processes and whether hypotension, as an additional insult, exacerbates brain injury or neuronal injury by changing the expression of mGluRs in the cerebral cortex, we studied the alteration of neuronal expression of mGluRs after DBI and DBI coupled with SBI in a rodent diffuse brain injury (DBI) model.

## Results

### Mortality and physiological Parameters of rats

No rats died in the normal, sham-operated control and SBI alone groups. Twelve of sixty rats in the DBI group died between 15 minutes and 1 hour after injury. Twenty-three of seventy-one rats in the DBI with SBI group died between 1 and 15 hours after injury. Compared with the DBI alone group, the DBI with SBI group showed higher mortality (P < 0.05 Table 1). We also monitored the physiological parameters of the rats 1 hour after surgery. There were no changes among groups (P > 0.05, Table 1) in the measured physiological parameters including mean arterial blood pressure (MABP), core temperatures, blood gas analysis, and blood glucose.

**Table 1:** Physiological Parameters 1 hour after experiment or injury (x¯
 MathType@MTEF@5@5@+=feaafiart1ev1aaatCvAUfKttLearuWrP9MDH5MBPbIqV92AaeXatLxBI9gBaebbnrfifHhDYfgasaacPC6xNi=xH8viVGI8Gi=hEeeu0xXdbba9frFj0xb9qqpG0dXdb9aspeI8k8fiI+fsY=rqGqVepae9pg0db9vqaiVgFr0xfr=xfr=xc9adbaqaaeGacaGaaiaabeqaaeqabiWaaaGcbaGafeiEaGNbaebaaaa@2D62@ ± s) and mortality of rats (%) in different groups

Group	No. Rats	(AMBP mmHg)	Core Temp (°C)	PaO2 (mmHg)	PaCO2 (mmHg)	PH	SaO2 (%)	Blood sugar (mmol/L)	No. Death	Percentage of Death (%).
Normal control	60	108.7 ± 10.4	37.3 ± 0.8	240.6 ± 30.8	39.4 ± 6.2	7.5 ± 0.1	99.5 ± 0.8	5.2 ± 0.2	0	0
Sham-operated	60	107.2 ± 12.9	37.1 ± 0.8	250.9 ± 25.3	37.2 ± 4.5	7.5 ± 0.2	99.3 ± 1.2	5.3 ± 0.3	0	0
SBI alone	60	110.5 ± 14.9	37.2 ± 0.6	247.5 ± 16.2	39.5 ± 7.4	7.4 ± 0.1	99.6 ± 2.3	5.5 ± 0.7	0	0
DBI alone	60	100.2 ± 16.2	37.7 ± 0.7	234.9 ± 21.8	40.3 ± 8.5	7.4 ± 0.2	99.4 ± 2.2	6.1 ± 0.4	12	20.0
DBI with SBI	71	103.6 ± 13.0	37.2 ± 0.3	226.6 ± 17.9	38.0 ± 6.8	7.3 ± 0.2	98.8 ± 4.3	5.6 ± 0.6	23	32.4 ^a^

^a ^P < 0.05 vs DBI alone group

The table shows the physiological parameters of the rats 1 hour after surgery. There were no changes among groups (*P *> 0.05) in the measured physiological parameters during the experiment, including MABP, core temperatures, blood gas analysis, and blood glucose. And the table also shows that no rats died in normal, sham-operated control and SBI alone groups. Twelve and 23 of the rats died in DBI and DBI with SBI group respectively. Compared with that in DBI alone group, the mortality of rats in DBI with SBI group was higher (P < 0.05).

### Neurological severity score (NSS) of rats

**Figure 1:**
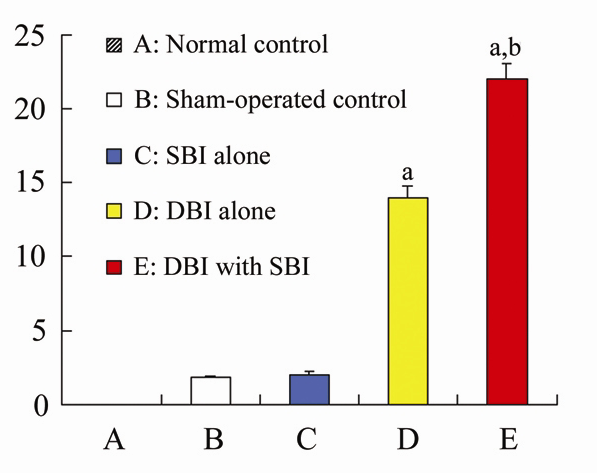
**Rat neurological severity score in different groups**. The bar graph shows that there were no statisticaldifferences in the NSS of rats among the normal, sham-operated control and SBI alone groups. The NSS of rats in the DBI alone and DBI with SBI group increased sharply compared with that of normal, sham-operated control and SBI alone groups. The NSS of rats in the DBI with SBI group also significantly increased compared with that in the DBI group. ^a^*P *< 0.05 *vs *normal, sham-operated control and SBI alone groups. ^b^*P *< 0.05 *vs *DBI alone group.

Between-group comparisons indicated that there were no statistical differences in the NSS of rats in the normal, sham-operated control and SBI alone groups (P > 0.05). The NSS of rats in the DBI alone and DBI with SBI group increased sharply compared with that of normal, sham-operated control and SBI alone groups (*P *< 0.05). The NSS of rats in the DBI with SBI group also significantly increased compared with that in the DBI alone group (*P *< 0.05, Figure 1).

### Expression of mGluRs

#### Group I mGluRs

**Figure 2:**
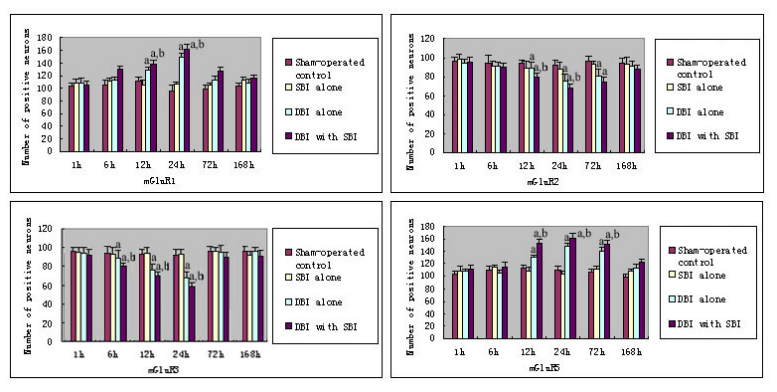
**Changes of mRNA of Group I and II metabotropic glutamate receptors after diffuse brain injury and diffuse brain injury with secondary brain insults. *Group I mGluRs ***The bar graph shows that there were no statistical differences in the number of group I and II mGluRs positive neurons among sham-operated control and SBI alone groups (*P *> 0.05). Compared with that of the sham-operated control and SBI alone groups, the number of group ImGluRs positive neurons increased at 12 hours and peaked at 24 hours in the DBI alone group (*P *< 0.05). And when compared with that in the DBI alone group, the number of group I mGluRs positive neurons increased significantly in the DBI with SBI group. In addition, the number of mGluR5 positive neurons remained higher until 72 hours (*P *< 0.05). In contrast to the expressing pattern of group I mGluRs, the number of mGluR2 positive neurons began to decrease 12 hours after injury and the lowest point occurred 24 hours after injury in the DBI alone group. The expression of mGluR3 significantly decreased 6 hours post-injuries and the lowest pointwas at 24 hours after injury (*P *< 0.05). In the DBI with SBI group, the changes in the number of mGluRs positive neurons were similar to that in the DBI alone group, but the number was much lower (*P *< 0.05). ^a^*P *< 0.05 sham-operated control and SBI alone groups. ^b^*P *< 0.05 *vs *DBI alone group.

There were no statistical differences in the number of group I mGluR positive neurons among normal, sham-operated and SBI alone groups (P > 0.05). In comparison with that of the normal, sham-operated control and SBI alone groups, the number of group I mGluR positive neurons increased at 12 hours and peaked at 24 hours in the DBI alone group (P < 0.05). When compared with that in the DBI alone group the number of group I mGluR positive neurons increased significantly in the DBI with SBI group. (P < 0.05, Figure 2).

#### Group II mGluRs

No statistical significance in the number of mGluR positive neurons among normal, sham-operated control and SBI alone groups was found (P > 0.05). In contrast to the expression pattern of group I mGluRs, the number of mGluR2 positive neurons began to decrease 12 hours after injury and the lowest point occurred 24 hours after injury in the DBI alone group. The expression of mGluR3 significantly decreased 6 hours post-injuries and the lowest point was at 24 hours after injury (P < 0.05). In the DBI with SBI group, the changes in the number of mGluR positive neurons were similar to that in the DBI alone group, but the number was much lower (P < 0.05, Figure 2)

#### Group III mGluRs

**Figure 3:**
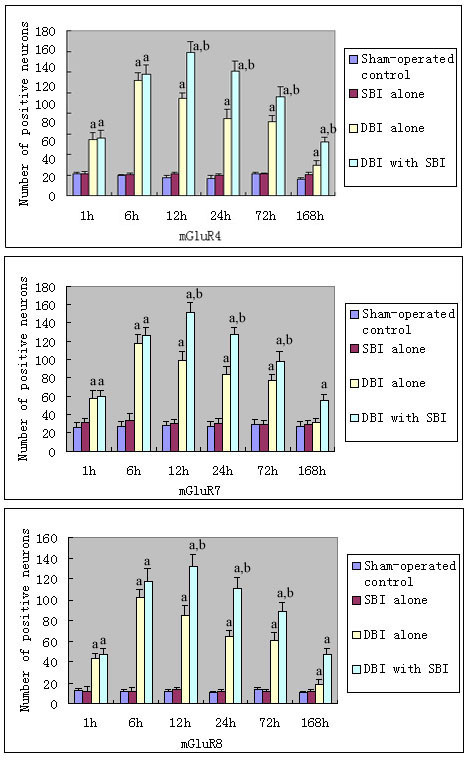
**Changes of mRNA of Group III metabotropic glutamate receptors after diffuse brain injury and diffuse brain injury with secondary brain insults**. The bar graph shows no statistical significance was found in the number of mGluR positive neurons among sham-operated control and SBI alone groups (*P *> 0.05). Compared with that in sham-operated control and SBI alone groups, the expression of mGluR4, 7, 8 in the DBI alone group increased significantly 1 hour after injury, and reached the highest level after 6 hours. The expression of mGluR4, 7, 8 in the DBI with SBI group was even higher than that in the DBI alone group. However, there were no significant changes in mGluR6 expression after injury. ^a^*P *< 0.05 *vs *sham-operated control and SBI alone groups. ^b^*P *< 0.05 *vs *DBI alone group.

**Figure 4:**
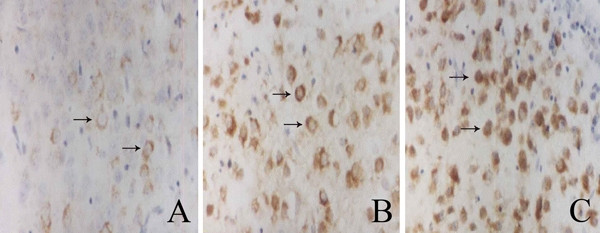
**Changes of mRNA of metabotropic glutamate receptor 4 after diffuse brain injury and diffuse brain injury with secondary brain insults**. **A: **in the sham-operated group, few mGluR4 positive neurons were found. **B: **the number of mGluR4 positive neurons increased and peaked 6 hours after DBI. C: the number of mGluR4 positive neurons in the DBI with SBI group was even higher than that in the DBI alone group. The arrows point at the positive neurons.

No statistical significance in the number of mGluR positive neurons were found among normal, sham-operated control and SBI alone groups (*P *> 0.05). In comparison with that in the normal, sham-operated control and SBI alone groups, the expression of mGluR4, 7, and 8 in the DBI alone group increased significantly 1 hour after injury, and reached the highest level after 6 hours (P < 0.05). The expression of mGluR4, 7, and 8 in the DBI with SBI group was even higher than that in the DBI alone group (P < 0.05, Figure 3, 4). However, there were no significant changes in mGluR6 expression after injury.

## Discussion

In the present study, the alteration of neuronal expression of mGluRs was studied on the Marmarou's impact acceleration model of closed head injury and the SBI model similar to that described by Ishige [24]. With the models, rats in the DBI with SBI group showed a higher mortality and NSS compared with that in the DBI alone group. The excess deaths in the DBI with SBI group were most likely due to the existence of posttraumatic SBI [25,26]. This result supports the hypothesis that posttraumatic SBI such as hypoxia and hypotension exacerbates neuronal injury and leads to worse outcome in TBI [13-16,27-29]. In fact, apart from TBI, the clinical outcome of many neurologic diseases in critical condition may also be partly determined by the number of SBI [30,31]. Therefore, research on posttraumatic SBI is of great value.

Our study suggested that mGluRs may be involved in the cellular processes after TBI and posttraumatic SBI. Glutamate is the principal excitatory neurotransmitter in the brain, and it was suggested that a wide variety of acute and chronic neurologic diseases may be mediated, at least in part, by a final common pathway of neuronal injury involving excessive stimulation of glutamate receptors [20,32]. However, due to the deficient properties of the molecules that entered human trials and to inappropriate design of clinical studies, glutamate N-methyl-D-aspartate receptor antagonists failed to show efficacy in clinical trials of stroke or TBI [33,34]. Therefore, the expression of mGluRs after TBI coupled with SBI should be studied to elucidate the mechanism and to find more powerful mGluRs receptor antagonists and agonists. In our present study, we systematically observed changes in the expression of the mGluRs after DBI and DBI with SBI in animals. Although we observed a significant elevation in the expression of group I and group III mGluRs (except mGluR6, represented by the number of mGluR4 positive neurons in figure 4) and a decrease in the expression of group II mGluRs in the supraventricular cerebral cortex after DBI, the changes were more severe when DBI was coupled with SBI. It was indicated that SBI may exacerbate neuronal injury or brain injury by altering expression of mGluRs, and more emphasis should be put on the prevention and treatment of SBI.

Our previous study investigated the alteration of expression patterns of mGluRs after DBI alone. We suggested that the damaged neurons utilize a self-protection first mechanism [17], because mGluR expression shows a characteristic time sequence (the damaged neurons begin to express protective group III mGluRs to protect themselves at first, and then express the neuro-harmful group I mGluRs), and the activation of group II and III mGluRs has been reported to be neuroprotective both in vitro and in vivo [35-37]. However, the expression of group II mGluR decreased when the expression of group I and III mGluRs increased in our study. The exact reason for this is not clear, but it is similar to Allen's study which suggested group II receptors are not activated sufficiently in response to trauma to provide endogenous neuroprotection [38]. Iversen's study even observed a decreased mRNA levels for mGluR1, mGluR2, mGluR5 and an unchanged mRNA level for mGluR3 in regions of rat brain after transient global ischemia [39]. After all, the present study further supports our previous suggestion in much more severe circumstances such as DBI coupled with SBI. It was indicated that a self-protection first mechanism of the damaged neurons was a spontaneous response of the neurons to trauma and was closely related to the severity of the brain damage. In our present study, the effect of protective group III mGluRs was followed by the more powerful effect of neuro-harmful group I mGluRs, which may initiate or augment the biochemical cascades [40]. Conceivably, the overall neuro-harmful effects of mGluRs and the following destructive cascades on neurons might be closely associated with the higher mortality and NSS of rats in the DBI with SBI group. Although this needs to be further clarified, it may provide clues for the understanding of the mechanism of DBI with SBI. The antagonists of group I mGluRs and agonists of group II and III mGluRs may provide neuroprotection against such brain injuries in selected conditions [41,42].

## Conclusion

The results of the present study suggest posttraumatic SBI exacerbates neuronal injury or brain injury by altering expression of mGluRs, and more emphasis should be put on the prevention and treatment of SBI.

## Methods

### Animal models and grouping

Three hundred and eleven male Sprague-Dawley rats (350 ± 15 g) entered our study. The surgical procedures followed the Fourth Military Medical University's guidelines for animal care and experiments. The rats were randomly assigned into five groups: normal control (A, 60 rats); sham-operated control (B, 60 rats); SBI alone (C, 60 rats); DBI alone (D, 60 rats); DBI with SBI (E, 71 rats). The five groups were studied at different time points: 1, 6, 12, 24, 72, and 168 hours post-injury (10 rats per time point). The rats were anaesthetized with 5.0% halothane induction and maintained at 1.5% halothane during surgery and throughout the brain injury procedures. An endotracheal tube was inserted orally so that the rats could be mechanically ventilated. A polyethylene cannula was inserted in the right femoral artery and the MABP was monitored via the artery and recorded for 60 minutes following injuries. Blood gases were obtained from arterial samples taken from the right femoral artery just before injury and again 60 minutes later. Blood glucose levels were also monitored. The DBI procedures were performed according to Marmarou's method [43] with minor modifications, summarized briefly as follows. After the exposure of the central area of the skull vault between the coronal and lambdoid sutures, a stainless-steel disc (1 cm diameter) was firmly fixed by dental acrytic to this central portion of the skull vault to disperse the strength of striking. Then the rat was placed on a foam bed in the prone position right under a 2-m-tall Plexiglas tube. A 450-g weight inside the tube was allowed to precisely strike the disc cemented to the skull face. The foam bed together with the rat was then moved away from underneath the tube immediately after the impact to insure a single hit. The SBI model was made as follows: the animals were calm for 15 minutes after anesthesia in the SBI alone group or after DBI. The right femoral artery and vein were cannulated for withdrawal and injection of blood while monitoring the MABP minute by minute. Rectal temperature was monitored continuously and maintained normothermic (37.5 ± 0.5°C) by use of an underbody heating blanket and a proportional feedback temperature controller (Harvard Homeothermic Blanket Control Unit). For animals in the DBI with SBI group, the MABP was maintained at 30 mmHg by withdrawing additional blood or by re-injecting blood during the 30 minute period of hypotension [24]. The animals were resuscitated by re-injecting blood and necessary fluids after the 30 minute period of hypotension. Then the animals were decapitated and sampled as scheduled.

### Behavior Examination

To observe the degree of injury, the neurological status of rats were evaluated just before death by NSS described by Shapira [44]. Table 2 summarizes the criteria for scoring. The NSS correlates directly with the deterioration of observable neurological status so that a lower score represents nearly intact neurological status and a higher score indicates severe neurological dysfunction caused by injury.

**Table 2:** Grading of neurological severity score of rats.

Behaviors			Points
Hemiplegia: inability resist to forced changes in position			1
Flexion in hindlimb when raised by tail			1
Inability to walk straight when placed on floor			1
Inability to walk			1
Reflex	Loss of righting reflex for	20 minutes	1
		40 minutes	1
		60 minutes	1
	Limb reflexes: loss of placing Reflexes	Left forelimb	1
		Left hindlimb	1
		Right forelimb	1
		Right hindlimb	1
Clinical grade	Loss of seeking behavior		1
	Prostration		1
Inability to exit from a circle (50 cm in diameter) when left in the center for Functioning test: failure in beam balancing task (1 cm wide)	20 minutes		1
	40 minutes		1
	60 minutes		1
	Balances with steady posture, paws on top of beam		1
	Grasps sides of beam and/or has shaky movement		1
	One or more paws slip off beam		1
	Attempt to balance on beam but falls off		1
	Drapes over beam and/or hangs on beam and falls off		1
	Falls off beam with no attempt to balance or hang on		1
Failure in beam walking task	2.5-cm wide		1
	5.0-cm wide		1
	8.0-cm wide		1
Sum			25

The table summarizes the criteria for scoring of rats. The NSS correlates directly with the deterioration of observable neurological status so that a lower score represents nearly intact neurological status and a higher score indicates severe neurological dysfunction caused by injury.

### mGluRs Study

To detect the mRNA of mGluRs in brain tissue, in-situ-hybridization was employed. At each time point described above, the animals were deeply anaesthetized with halothane and decapitated. The brains were extracted and fixed with a solution of paraformldehyde (dissolved in pyrocarbonic acid diethyl ester water) for 6–8 hours. Then the brains were sectioned in coronal planes and embedded in paraffin. The injured supraventricular cerebral cortex between the coronal and lambdoid sutures was sampled. These areas were just under the stainless-steel disc. Our previous study indicated that the pathological changes in this area were more obvious than that in other parts of the brain [17]. Six μm thick coronal consecutive sections were obtained with a rotary microtome and one of every ten sections was chosen to undergo the hybridization in situ procedure, using the hybridization in situ kit from WuHan boster Biological Technology Co. The oligonucleotide probe modified with digoxin at the 5' end was synthesized by Shanghai Bioasia Co. The probe sequences are as follows:

mGluR1 (5'-CCCCACGTGGACATAGTCATAGCGATTAGCTTCTGTGTACTGCAG-3')

mGluR2 (5'-GGTGACAGCTGTAGGAGCATCACTGTGGGTGGCATAGGAGCCATC-3')

mGluR3 (5'-CTGAGAATAGGTGGTTGCAGTTCCGCTGACGCTGAACCTGTTGAG-3')

mGluR4 (5'-GGTCTCCAGGTTCTCACACAGCTCTGATTTGGCTTCCCCATTGGG-3')

mGluR5 (5'-GGA GCG GAA GGA AGA AGA TCC ATC TAC ACA GCG TAC CAA ACC TTC-3')

mGluR6 (5'-TTCTCAGTGCAGCCTCCCCTGCGGGCCCGGTGAACGGAAAAAGAT-3')

mGluR7 (5'-CTTCTTTTTTGCAGCAGGGCTGTTTGGGTCTACATTTTCACAGAG-3')

mGluR8 (5'-ATGTTGTCTTGGTAGAAGAAGTGTTGGTTTCAAGACTCTCACAGA-3').

All the probe sequences were analyzed on the Basic Local Alignment Search Tool (BLAST) of the National Library of Medicine. The probe for each mGluR hybridized well with the corresponding substrate, and showed no homology with other gene sequences. With these probes, the specificity of the signals obtained from in-situ hybridization can be confirmed.

To assess quantitatively the expression of mGluRs, the number of positive neurons on each of the chosen sections was counted manually in eight different fields which were randomly chosen under the high-power microscope (400×). The sample borders of the brain regions for assessment of the number of positive neurons were selected just under the stainless-steel disc between the coronal and lambdoid sutures as mentioned above. The lateral ventricle served as a landmark to locate the regions which were situated in the supraventricular area. All the sampled brain regions were of the same size for each animal. The positive neurons were stained brown in the cytoplasm, and sometimes in the nucleolus. Glial cells that also express mGluRs could be distinguished from neurons by their morphological difference. An investigator who was blind to the experimental plans performed all of the assessments.

### Statistics analysis

All the data were expressed as mean ± standard deviation. The mortality was analyzed by χ^2 ^test. The physiological parameters of the rats 1 hour after injury, the NSS of the rats, and the number of positive neurons were analyzed by ANOVA of SPSS 9.0 statistical software. P < 0.05 was considered statistically significant.

## List of abbreviations

BLAST: basic local alignment search tool;

CBF: cerebral blood flow;

DBI: diffuse brain injury;

iGluRs: ionotropic glutamate receptors;

MABP: mean arterial blood pressure;

mGluRs: metabotropic glutamate receptors;

NSS: neurological severity score;

SBI: secondary brain insults;

TBI: traumatic brain injury;

## Authors' contributions

Zhou Fei established all the animal models and prepared the first draft of the manuscript. Xiang Zhang carried out statistical analyses of the raw data, prepared the graphs and formatted the first draft. Hong-min Bai and Xiao-fan Jiang performed the behavior examinations for experimental animals. Xia Li performed the in-situ hybridization for all sections. Wei Zhang and Wei Hu accessed quantitatively the expression of mGluRs. All authors read and approved the final manuscript.
